# From believers to skeptics: Latent class analysis of COVID‐19 protective practices and perceptions among agricultural community members

**DOI:** 10.1111/jrh.12692

**Published:** 2022-07-16

**Authors:** Josie M. Rudolphi, Courtney Cuthbertson, Amandeep Kaur, Jesus N. Sarol

**Affiliations:** ^1^ Department of Agricultural and Biological Engineering University of Illinois Urbana‐Champaign Urbana Illinois USA; ^2^ Department of Human Development and Family Studies University of Illinois Urbana‐Champaign Urbana Illinois USA; ^3^ Interdisciplinary Health Science Institute University of Illinois Urbana‐Champaign Urbana Illinois USA

**Keywords:** agriculture, COVID‐19, latent class analysis, occupation, rural

## Abstract

**Purpose:**

This cross‐sectional study aimed to identify homogenous groups of agricultural producers and stakeholders based on their perceptions of effectiveness and use of COVID‐19 protective behaviors.

**Methods:**

We conducted an online survey of agricultural producers and stakeholders through Qualtrics. Participants responded to 7 statements about COVID‐19 protective behavior effectiveness and 7 statements about participation in COVID‐19 protective behaviors in the previous 2 weeks. These statements included handwashing, disinfecting, refraining from touching one's face, covering one's face when coughing/sneezing, staying at home, social distancing, and wearing a face mask. Additional survey sections included demographics and health history. We performed separate latent class analysis (LCA) to identify clusters of agricultural producers’ and stakeholders’ perceptions and participation in COVID‐19‐related protective behaviors based on their pattern of responses.

**Findings:**

Based on LCA, participants were distributed as universal believers (33%), social believers (16%), personal believers (26%), moderate believers (17%), and social skeptics (85%) of effectiveness and as low (15%), moderate (40%), and high (45%) adherents of COVID‐19 protective behaviors. Those who were female, older, or had underlying health conditions were more likely to be universal believers and highly adherent. High adherence was also more likely among those who lived in urban areas or were not self‐employed.

**Conclusions:**

Results suggest that groups of agricultural producers and stakeholders based on perception of effectiveness and participation in COVID‐19 protective behaviors are associated with demographic and health characteristics. Public health campaigns that increase or maintain motivation to comply with protective behaviors should be developed and implemented specific for agricultural populations.

## INTRODUCTION

The COVID‐19 pandemic as a public health emergency has required mass engagement in protective behaviors, such as staying at home, social distancing, wearing face masks, and frequent handwashing, in order to curb the spread of the potentially fatal virus.^1^ However, COVID‐19 risks have not been distributed evenly across the population. Lower geographic density and isolation common to rural areas may have initially reduced risk of COVID‐19 in rural areas, by March 2021 COVID‐19 spread was higher rural areas.^2^, ^3^ COVID‐19 vaccination rates have been lower in rural counties, and within rural counties, lowest among counties that are farming dependent.^4^ Additionally, people in occupations deemed essential were, at times, unable to engage in some protective behaviors based on the nature of their work.^5^

Jobs related to agriculture were deemed essential during pandemic in order to ensure that adequate food and other supplies were available to consumers, and those employed in agriculture faced higher risk of exposure to COVID‐19.^6^ Several factors may increase the risk of COVID‐19 for people in agricultural occupations. Tasks and activities at agricultural workplaces were not interrupted in response to COVID‐19. In the Midwest United States, time‐sensitive tasks, such as planting and harvesting continued, even when other businesses temporarily closed or shifted to work‐from‐home conditions.^1^ Production activities, such as planting and harvesting, may increase the risk of COVID‐19 transmission. Additionally, many agricultural producers and workers reside in rural areas, where rates of chronic diseases are higher.^7^, ^8^, ^9^, ^10^, ^11^ Furthermore, in the United States, recommendations, use, and beliefs in effectiveness of COVID‐19 protective behaviors have been contested. Skepticism in science,^12^ belief in conspiracy theories,^13^ conservative media use,^14^ and belief that the risk of COVID‐19 had been exaggerated^15^ have all been associated with less use of COVID‐19 protective behaviors. However, most of the studies are based on the general population, and information about how agricultural producers responded to COVID‐19 and COVID‐19 protective recommendations is needed.

The objective of this study was to describe how people in agricultural occupations perceive and engage in COVID‐19 protective behaviors, and which demographic and work factors were associated with reported protective behaviors. Analysis of COVID‐19 protective behavior participation and perceptions about their effectiveness can help to inform public health information dissemination strategies among this occupational group deemed essential.

## METHODS

This cross‐sectional study surveyed agricultural producers and stakeholders from online from April to June 2020 via Qualtrics. Study procedures were approved by the University of Illinois Urbana‐Champaign Intitutional Review Board. University of Illinois Extension and Illinois Farm Bureau shared information about the study in e‐newsletters, emails, and social media posts. Eligibility criteria included being age 18 or older, and self‐identifying as an agricultural producer, defined as *an individual actively engaged in the production of livestock, crops, or other commodities for sale*, and/or agricultural stakeholder, defined as *an individual whose occupation directly serves agricultural producers*. Participants were not compensated for completing the questionnaire. The survey included questions about self‐rated physical and mental health, whether participants had chronic health conditions associated with higher severity of COVID‐19 (chronic lung disease, severe to moderate asthma, serious heart condition, severe obesity, diabetes, chronic kidney disease, and liver disease), demographic characteristics, and farming/work characteristics. Respondents also indicated their age (≤64 years or ≥65 years), sex (male or female), education level (high school or G.E.D or less; technical, trade, associate degree; bachelor degree or higher), race (white or others), self‐employment status (yes, no, prefer not to disclose), and residence (urban, suburban, rural, or other). Respondents were only able to select 1 option for residence. Definitions for rural, urban, and suburban were not provided.

This article focuses on questions from the survey about perceived effectiveness of and participation in 7 COVID‐19 protective behaviors recommended by the Centers for Disease Control and Prevention (CDC; washing hands with soap regularly, disinfecting heavily used surfaces, staying at home as much as possible, practicing social distancing, covering cough or sneeze, wearing a mask, and refraining from touching eyes, nose, and mouth). Respondents indicated on a 3‐point Likert scale how effective (not at all effective to very effective) each of the 7 the CDC recommended protective measures are at reducing the spread of COVID‐19. Similarly, respondents indicated on a 5‐point Likert scale how often (never to every day) they practiced each of the 7 CDC‐recommended protective behaviors in the previous 2 weeks.

### Sample

Our sample consisted of 1,441 respondents who were mostly white (98%), men (82%), self‐employed (74%), resided in rural areas (89%), and identified as agricultural producers (85%). The majority had obtained a bachelor's degree (54%), while another 24% had technical, trade, or associate degrees. Age ranged from 18 to 93 years (mean = 58.0, SD 14.3), where 64% were 64 years or under.

The vast majority of respondents were from [State] (97%). [State] is a major producer of soybeans, corn, and pigs, and agriculture contributes over 50 billion dollars to the state's economy annually.^16^ Approximately 6% of [State]’s workforce is in agriculture, as agriculture employs nearly 450,000 individuals, including 116,417 agricultural producers.^17^ The sample of the current study is very similar to the agricultural producer population in the United States, which is 95% white, majority men (64%), and a mean age of 57.5 and is more similar to the producer population in [State], which is 99% white, nearly three‐quarters men (71%), and a mean age of 58.0.^17^

### Statistical analysis

Latent class analysis (LCA) was used to identify homogeneous, mutually exclusive agricultural groups based on perceived effectiveness and reported participation in COVID‐19 protective behaviors. LCA is a statistical method to identify clusters of participants based on shared characteristics.^18^ Latent classes have been used to identify health behavior patterns within heterogeneous populations,^19^ to identify patterns of compliance with behavioral recommendations to contain COVID‐19,^20^ and perceptions of threat and confidence during COVID‐19.^21^ LCA was performed using PROC LCA in SAS 9.4.^22^

Separate LCA for perceptions and practices of COVID‐19‐related protective behaviors was conducted. We fitted LCA models from 2 to 8 clusters. To ensure that the global maximum likelihood was obtained, we specified 50 random starting values (seeds) to obtain separate estimates of a model. The fit indices (Akaike information criterion [AIC], consistent Akaike information criterion [CAIC], Bayesian information criterion [BIC], and sample‐size adjusted Bayesian information criterion [SABIC]) were used for the selection of the optimal number of clusters. Interpretability of clusters and parsimony were also considered in the selection. The estimated probabilities (proportions) of producers and stakeholders belonging to each cluster as well as the conditional probabilities of the categories of the observed variables in each cluster were derived. The conditional probabilities were used to interpret the latent clusters. We tested the latent class models for measurement invariance across subgroups of gender, age, occupation, self‐employment, rural residence, and presence of underlying health conditions.

Bayesian posterior probabilities of membership of an individual to the latent clusters were generated in LCA. We then assigned each agricultural producer and stakeholder to the cluster of perception of effectiveness and practice based on maximum posterior probability of membership. We assessed goodness of fit by examining entropy and the average latent class posterior probabilities (ALCPPs). Using the categorization to latent classes, chi‐square tests were conducted to test the association of perceptions of effectiveness and practices of COVID‐19‐related protective measures. Similarly, chi‐square tests were used to examine the association of perceptions and practices with demographic variables and health conditions. Fisher's exact test was conducted when the chi‐square test assumptions were violated. Last, using single‐step approach, where covariates are included in determining latent classes, separate multinomial logistic regression analyses were conducted to determine which of the demographic variables and presence of comorbidity are strong predictors of latent classes of perceptions of effectiveness and practice of COVID‐19‐related protective measures.^23^ We used the *P*‐value of .25 in the bivariate association as the cutoff for statistical significance for inclusion of covariates in the model.

## RESULTS

Through LCA, we were able to determine distinct clusters based on participant responses to perception of effectiveness and practice of specific COVID‐19 protective behaviors. Based on fit indices (CAIC and BIC), parsimony, and interpretability, 5 clusters for perceptions of effectiveness and 3 clusters for practice of COVID‐19 protective behaviors were identified (Table 1 and Table A1). Based on group responses to survey questions, we provided these cluster labels for perception of effectiveness: (1) universal believer; (2) personal believer; (3) social believer; (4) moderate believer; (5) social skeptic; and for practices of COVID‐19 protective behaviors: (1) high adherents; (2) moderate adherents; and (3) low adherents.

**TABLE 1:** Distribution of agricultural producers and stakeholders according to membership in participation in COVID‐19 protective behaviors cluster, by membership in perception of effectiveness cluster

	Perception of effectiveness of COVID‐19 protective behavior cluster				
Practice of COVID‐19 protective behavior cluster	Universal believer	Personal believer	Social believer	Moderate believer	Social skeptic
High adherent	334 (72.3)	154 (39.8)	109 (46.0)	49 (22.0)	7 (5.7)
Moderate adherent	118 (25.5)	192 (49.6)	105 (44.3)	121 (54.3)	44 (35.8)
Low adherent	10 (2.2)	41 (10.6)	23 (9.7)	53 (23.8)	72 (58.5)
	Over‐all χ^2^=434.54 (*P*<.0001)				

### Perceptions of effectiveness of COVID‐19 protective behaviors

The largest cluster comprised of 33% of producers and stakeholders who predominantly perceived that all the COVID‐19 protective measures are very effective (universal believer). A very significant distinction of this cluster from the others is that the majority of universal believers said wearing a face mask is very effective. The second largest cluster (26%) of producers and stakeholders were primarily those who considered personal measures (washing hands, disinfecting, refrain from touching the face, and cover cough and sneeze) are very effective but believed social measures (staying home, social distancing, and wearing a face mask) are only somewhat effective (personal believers). The third cluster comprised of 16% of producers and stakeholders, where the majority perceived social measures, such as staying home and social distancing, to be very effective (social believers). Large proportions in this cluster also perceived certain personal measures (washing hands and covering cough and sneeze) to be very effective. Other COVID‐19‐related personal preventive measures and wearing a face mask were rated as only somewhat effective by most social believers. The fourth cluster of producers and stakeholders (17%) considered all COVID‐19 protective behaviors as only somewhat effective. We referred to them as moderate believers. The fifth and smallest cluster (8%) are described as social skeptics who greatly perceived social measures as ineffective and personal measures as only somewhat effective except for covering cough and sneeze (Table A1).

### Practices of COVID‐19 protective behaviors

LCA of COVID‐19 preventive practices showed 3 clusters as the best fit based on both CAIC and BIC indices. When asked how often they practiced COVID‐19‐related protective behaviors in the past 2 weeks, the largest cluster of producers and stakeholders (45%) were in the risk‐averse or high adherence group. The majority in this group reported that they observed all personal and social measures every day and always wore a face mask when going out in public. In contrast, 15% of participants are described as low adherents. Over half of this group reported that they never practiced disinfecting surfaces, refraining from touching their face, or wearing face masks. The majority of producers and stakeholders in this group either never stayed at home or only for a few days in the past 2 weeks. However, washing hands and covering cough and sneeze was practiced by most producers and stakeholders in this cluster. The third group comprising of 40% producers and stakeholders are labeled as moderate adherents. Washing hands, refraining from touching their face, covering cough and sneeze, social distancing, and staying at home were reported to be practiced daily or for most days in the past 2 weeks. However, the majority said they practiced disinfecting for half of the days or less. Only 20% always wore a face mask when in public and the rest did so half of the time or less (Table A1).

### Measurement invariance of latent classes

We compared fit indices of free‐parameter models and measurement invariant models across categories of gender, age, occupation, self‐employment, residence (urban‐rural), and presence/absence of underlying health conditions. Based on BIC, the measurement invariant models provided a better fit of the data (Table A2). This result meant that the latent classes have similar interpretations in the different subgroups.

### Association between perceptions of effectiveness and practices of COVID‐19 protective practices in the past 2 weeks

We categorized each producer and stakeholder to the perception of effectiveness and practice cluster where they are most likely to be a member as determined by Bayesian posterior probabilities. The entropy for the LCA models was 0.78 for perception of effectiveness and 0.74 for practice. These values are near 0.8, a widely accepted level entropy value for good fit.^24^ We found that the ALCPP was 0.867 for perception of effectiveness clusters and 0.879 for participation clusters. These results indicated that the individuals were most likely grouped into the correct clusters using maximum‐probability assignment. Chi‐square tests showed a significant association between perceptions of effectiveness and use of protective practices (*P*<.0001). While 72% of universal believers were in the high adherence group, only 40% of personal believers, and 46% of social believers were in the high adherence group. Comparatively, 50% of personal believers, 44% of social believers, and 54% of moderate believers were in the moderate adherence group. Last, 59% of social skeptics were in the low adherence group (Table 2).

**TABLE 2:** Chi‐square tests for pairwise comparisons of distribution of agricultural producers and stakeholders according to cluster membership in participation in COVID‐19 protective behaviors between clusters of perception of effectiveness

	Personal believer	Social believer	Moderate believer	Social skeptic
	χ^2^ (*P*‐value)	χ^2^ (*P*‐value)	χ^2^ (*P*‐value)	χ^2^ (*P*‐value)
Universal believer	97.0329 (<.0001)	53.2491 (<.0001)	179.9847 (<.0001)	297.8181 (<.0001)
Personal believer	–	2.3235 (0.3129)	30.0262 (<.0001)	135.0696 (<.0001)
Social believer	–	–	35.3663 (<.0001)	115.4095 (<.0001)
Moderate believer	–	–	–	45.1948 (<.0001)

### Association of perceptions of effectiveness and practices of COVID‐19 protective behaviors in the past 2 weeks with demographic characteristics, work factors, and health conditions

While the association of perceptions of effectiveness of COVID‐19 protective behaviors was found to be statistically significant with sex (*P*<.0001), occupation (*P*=.0122), age (*P*<.0001), and having diabetes (*P*=.0014) (Table 2), the COVID‐19 reported protective practices were found to be statistically significant with sex (*P*<.0001), self‐employment (*P*=.0483), age (*P*<.0001), residence (*P*=.0043), chronic lung disease (*P*=.0104), moderate to severe asthma (*P*=.0359), and serious heart condition (*P*=.0217) of the producers and stakeholders (Table 3). Among universal believers, majority were males (74%), agricultural producers (88%), under the age of 64 years (52%), and nondiabetic (89%). Similarly, among social skeptics, the majority were males, agricultural producers, below the age of 64 years, and nondiabetic. The same trend was also seen for personal, social, and moderate believers. Among those who highly adhered to the COVID‐19 protective measures, about 75% were males, 56% were under the age of 64 years, 86% resided in rural areas, and about 95% had no chronic lung disease, moderate to severe asthma, or serious heart condition. Similarly, among those who had low adherence to COVID‐19 protective measures, the majority were also males (94%), under the age of 64 years (74%), lived in rural areas (93%), had no chronic lung disease (99%), asthma (99%), or serious heart condition (96%). The distributions of producers and stakeholders by education were similar across clustering of respondents based on perception of effectiveness (*P*=.3908) and practice (*P*=.9430) of COVID‐19 protective behaviors (Table 3).

**TABLE 3:** Association of perceptions of effectiveness of and participation in COVID‐19 protective behaviors with demographic and health profile

		Perceptions of effectiveness COVID‐19 protective behaviors					COVID‐19 reported protective practices in past 2 weeks			
Variable	Category	Universal believer (n=457)	Personal believer (n=383)	Social believer (n=235)	Moderate believer (n=219)	Social skeptic (n=123)	High adherent (n=549)	Moderate adherent (n=577)	Low adherent (n=200)	
Sex	Male	73.5	78.3	87.7	91.8	92.7	74.6	85.6	94.0	
	Female	26.5	21.7	12.3	8.2	7.3	25.4	14.4	6.0	
		*P*<.0001					*P*<.0001			
Age	≤ 64 years	51.5	74.9	54.4	67.3	85.4	56.4	68.4	73.8	
	≥ 65 years	48.5	25.1	45.6	32.7	14.6	43.6	31.6	26.2	
		*P*<.0001					*P*<.0001			
Education	High school, G.E.D, or less	22.5	19.3	21.5	24.2	26.0	21.1	22.5	23.8	
	Technical, trade, associate degree	22.5	27.7	26.6	19.7	22.0	24.5	23.7	23.8	
	Bachelor degree or higher	55.0	53.0	51.9	56.1	52.0	54.4	53.8	52.5	
		*P*=.3908					*P*=.9430			
Race	White	98.7	97.9	97.0	99.6	97.5	97.5	99.1	98.0	
	Others	1.3	2.1	3.0	0.5	2.5	2.5	0.9	2.0	
		*P*=.2039^a^					*P*=.1018			
Occupation	Agricultural producer	87.9	81.4	86.5	83.0	90.2	84.9	85.4	86.6	
	Agricultural stakeholder	10.6	13.2	9.7	13.9	4.9	11.9	10.5	10.9	
	Both	1.5	5.5	3.8	3.1	4.9	3.2	4.1	2.5	
		*P*=.0122					*P*=.7268			
Self‐employed	Yes	72.6	67.5	76.1	71.7	69.9	68.5	73.1	76.6	
	No	24.5	29.4	20.9	23.7	27.6	27.7	24.6	19.4	
	Prefer not to disclose	2.9	3.2	3.0	4.6	2.4	3.7	2.3	4.0	
		*P*=.1706^b^					*P*=.0483^b^			
Residence	Urban	3.1	3.3	2.1	3.8	3.6	1.6	4.4	1.9	2.0
	Suburban	7.4	7.8	7.8	7.2	7.2	6.5	9.3	6.4	4.5
	Rural	88.8	88.7	89.4	87.3	88.8	91.1	85.7	90.9	93.1
	Other	0.7	0.2	0.8	1.7	0.5	0.8	0.6	0.9	0.5
		*P*=.9201^b^					*P*=.0043^b^			
Have chronic lung disease	Yes	3.5	5.0	3.9	2.1	2.2	1.6	5.0	2.4	1.5
	No	96.5	95.0	96.1	97.9	97.8	98.4	95.0	97.6	98.5
		*P*=.1442					*P*=.0104			
Have moderate to severe asthma	Yes	4.4	5.6	3.9	3.8	4.5	2.4	5.2	4.6	1.0
	No	95.6	94.4	96.1	96.2	95.5	97.6	94.8	95.4	99.0
		*P*=.5183					*P*=.0359			
Have serious heart condition	Yes	4.4	6.5	2.6	5.1	3.1	3.3	6.0	2.7	4.0
	No	95.6	93.5	97.4	94.9	96.9	96.8	94.1	97.3	96.0
		*P*=.0535					*P*=.0217			
Have severe obesity	Yes	3.5	3.0	3.4	4.2	4.0	3.3	3.4	4.0	2.5
	No	96.5	97.0	96.6	95.8	96.0	96.8	96.7	96.1	97.5
		*P*=.9244					*P*=.6016			
Have diabetes	Yes	8.6	11.5	5.2	7.2	12.6	4.9	10.4	7.7	5.5
	No	91.4	88.5	94.8	92.8	87.4	95.1	89.6	92.3	94.6
		*P*=.0014					*P*=.0569			
Have chronic kidney disease	Yes	1.6	2.6	1.0	2.1	0.9	0.0	1.8	1.7	0.5
	No	98.4	97.4	99.0	97.9	99.1	100.0	98.2	98.3	99.5
		*P*=.1815^a^					*P*=.3987			
Have liver disease	Yes	0.6	1.1	0.0	0.8	0.9	0.0	0.6	0.9	0.0
	No	99.4	98.9	100.0	99.2	99.1	100.0	99.4	99.1	100.0
		*P*=.1894^a^					*P*=.5337^a^			

*Note*: Numbers in cells are percentages.

All *P*‐values are calculated with chi‐square test of independence.

^a^ Fischer exact test was used where chi‐square test failed.

^b^ Chi‐square was calculated after excluding the “Other” category in Residence and “Prefer not to disclose” in self‐employed.

Multinomial logistic regression was conducted to determine which of the demographic and health characteristics were associated with perception of effectiveness and practice of COVID protective behaviors. Gender, age, type of farmer, self‐employment, urban‐rural residence, and presence of comorbidities were included in the model, while educational attainment was not since it did not reach the cutoff of *P*=0.25 in the bivariate association. Race was not included because more than 98% of participants were white. Age was treated as a continuous variable, while a new variable of existing health condition was created based on the presence of 1 or more physical health conditions, coded as 1 for any existing health condition present and 0 if none. The universal believers and high adherent groups were selected as reference categories for respective cluster outcomes, perception of effectiveness, and practice of COVID‐19 protective behaviors. The clustering of producers and stakeholders based on perception of effectiveness of COVID‐19 measures is strongly associated with gender and age (*P*<.0001). The odds of being social skeptics and moderate believers relative to being universal believers are 6.76 (95% CI: 2.62‐17.45) and 4.80 (95% CI: 2.55‐9.04) times, respectively, in male respondents compared to female respondents. Male respondents also have higher odds than female respondents of being personal believers and social believers relative to universal believers, but to a lesser extent (OR=1.55 [95% CI: 1.02‐2.36] and 3.93 [95% CI: 1.67‐9.28], respectively). The odds of being social skeptics, moderate believers, or personal believers than being universal believers decrease with age (OR=0.51 [95% CI: 0.42‐0.62], 0.81 [95%CI: 0.70‐0.95], and 0.61 [95% CI: 0.52‐0.71], respectively, for every 10‐year age difference). After controlling for other factors, perceptions of effectiveness of COVID‐19 protective behaviors were found to be associated with agricultural occupation (*P*=.0645). Agricultural producers have odds of being social skeptics to being universal believers that are 3.94 (95% CI: 1.16‐13.41) times that for agricultural stakeholders. This odds ratio is markedly increased (OR=6.94, 95% CI: 1.21‐39.89) when the respondent is both a producer and a stakeholder compared to solely being a stakeholder. The odds of social believers and personal believers relative to universal believers among both producers and stakeholders were, respectively, 3.19 (95% CI: 0.73‐14.01) and 2.25 (95% CI: 0.69‐1.61) times that for stakeholders only. However, the odds ratios of social believers, personal believers, and moderate believers relative to universal believers comparing agricultural producers to agricultural stakeholders were lower, although these results did not clearly rule out incompatibility with having similar odds (Table A3).

Likewise, gender and age were strongly associated with the grouping of respondents based on their practice of COVID‐19 protective behaviors (*P*<.0001). The odds of being low adherents and moderate adherents relative to being high adherents were, respectively, 12.69 (95% CI: 3.44‐46.84) and 3.21 (95% CI: 2.05‐5.02) times as large in male farmers compared to female farmers. The odds ratios for 10‐year difference in age were 0.67 (95% CI: 0.58‐0.79) and 0.69 (95% CI: 0.61‐0.78), respectively, for low adherence and moderative adherence relative to high adherence for older agricultural producers and stakeholders. After controlling for other factors, presence of underlying health conditions was moderately associated with participation in COVID‐19 protective behaviors (*P*=.0243). Respondents who had underlying health conditions had lower odds of being low adherents (OR=0.50, 95% CI: 0.26‐0.93) and moderate adherents (OR=0.72, 95% CI:0.49‐1.04) relative to being high adherents compared to those without health conditions. Weaker association with self‐employment (*P*=.0573) and residence (*P*=.1025) was found. Compared to other producers and stakeholders, self‐employed had odds of low adherence 1.95 (95% CI: 1.08‐3.52) and of moderate adherence 1.23 (95% CI: 0.85‐1.79) times higher odds relative to high adherence. The odds of being low adherents and moderate adherents relative to being high adherents among rural residents were, respectively, 2.33 (95% CI: 0.89‐6.10) and 1.35 (95% CI: 0.81‐2.26) times as large as that of those living in urban areas (Table A4).

## DISCUSSION

LCA clarified discernable differences between agricultural groups based on their perceptions of effectiveness and participation in COVID‐19 protective behaviors. Most respondents participated in COVID‐19 protective measures with moderate or high adherence and over half of respondents were either universal believers or personal believers in the effectiveness of COVID‐19 protective measures. Notable associations between perceived effectiveness of and participation in COVID‐19 protective measures emerged. Over 70% of universal believers were in the high adherence group. Conversely, 54% of moderate believers were in the moderate adherence group and 59% of social skeptics were in the low adherence group. Engaging in protective behavior is associated with beliefs that such behaviors are effective to prevent COVID‐19; even moderate beliefs in effectiveness are helpful toward engaging more frequently in protective behaviors. However, a majority of respondents were from [State], and as of April 30, 2020, an Executive Order in the state required all individuals to wear a mask or face‐covering in indoor public places.^25^ Such mandates encourage, or even require, participation in COVID‐19 protective behaviors regardless of one's perceptions of effectiveness.

Interestingly, a higher percentage of respondents who were in the social believers group were in the high adherence group compared to the personal believers. This suggests a stronger willingness to look out for community members by participating in socially protective behaviors, than for oneself by participating in individually protective behaviors. This socially protective attitude may be explained by environmental norms or social values. Residents of rural communities, where most of the agricultural work still occurs, tend to place emphasis on taking care of family and kinfolk, and hold values related to the responsibility for others’ wellbeing.^26^, ^27^ Other studies using LCA related to COVID‐19 protective behaviors have also found 3 groups related to adherence or compliance, of high; public, mixed, or moderate; and low.^20^, ^28^, ^29^ Our study confirms that those who had strong beliefs in the effectiveness of protective behaviors were less likely to be in a low adherence group.^28^ However, additional research should explore the causal relationship between perceived effectiveness and participation in COVID‐19 protective measures, which could inform targeted public health communication campaigns to focus on either increased perceived effectiveness or participation in protective measures.

Across the 5 perception groups, washing hands and covering a cough or sneeze were perceived as very effective COVID‐19 protective behaviors among our sample of agricultural producers and stakeholders, whereas wearing a face mask and staying home were perceived as not effective. Although recommended by the CDC, face masks are consistently considered not effective by the general populations at preventing transmission of COVID‐19.^30^, ^31^ In a sample of US and UK survey participants, only 38% agreed that “*consistently wearing a face mask is highly effective in protecting you from getting infected with the new coronavirus*.”^30^ Conversely, and congruent with results from our sample, over 90% of perceived washing hands and avoiding touching one's eyes, nose, and mouth were effective at preventing COVID‐19 infection.^30^ Additional research should examine discrepancies in perceived effectiveness between evidence‐based COVID‐19 protective behaviors, and public health campaigns should target misinformation and misconceptions about COVID‐19 protective practices that are not considered effective among the agricultural producer and stakeholder population.

Across all 3 adherence groups, washing hands and covering cough were among the protective measures most reported. This aligns with findings from Germany that even individuals in a low compliance group participated in covering a cough or sneeze.^20^ These behaviors are often communicated and encouraged annually during influenza seasons and commonly practiced among adults in the United States.^32^, ^33^

Wearing a face mask yielded the most disagreement between the adherence groups. About 60% of agricultural producers and stakeholders in the high adherence group reported wearing a face mask every day, whereas less than 10% of producers and stakeholders in the low adherence group reported wearing a face mask with the same consistency. Despite their effectiveness in reducing the spread of COVID‐19, many people in the United States have been resistant to wearing face masks,^34^ and rural residents are much less likely to do so.^35^

Women and individuals 65 years of age or greater were more likely to be in the high adherence group. These trends have been observed in previous studies of COVID‐19 protective behaviors, where women were more likely to be wearing masks than men.^36^ Norms related to masculinity and demonstrating toughness have driven men to be more resistant to wearing face masks.^37^ Low compliance has been associated with being male and younger age,^20^, ^28^ whereas women and older adults consistently participate in preventative behaviors to mitigate health risks.^38^, ^39^ Women and older adult groups may perceive greater risk of COVID‐19; the CDC communicated early in the pandemic that risk of severe illness increases with age, which may have encouraged participation in protective measures among aging individuals and women have been found to perceive higher risk of infection when compared to men.^40^ Women are often socialized into caretaking roles, which include looking out for others, compared to men who are not expected to care for others. In response, women may be more concerned about the health and wellbeing of the people around them and more likely to adhere to CDC recommendations. This aligns with the results of Missourians’ preferences of measures to prevent the spread of COVID‐19, wherein men were twice as likely as women to be in the LCA group preferring keeping all services open, rather than being in the group holding strong preferences for all possible COVID‐19‐related restrictions.^41^ Additionally, women have had higher rates of vaccination in both urban and rural areas,^42^ which further supports their role as community caretakers.

Understanding perceptions and use of protective practices related to COVID‐19 in agricultural and rural communities is critical to informing public health intervention strategies. Rural communities are considered at‐risk by CDC as they often have a greater proportion of older adults, higher rates of chronic disease, higher rates of disabilities, and limited health care infrastructure.^43^ All of these factors, as well as higher rates of uninsured people in rural areas, drive one‐third of rural counties to be highly susceptible to COVID‐19.^44^ COVID‐19 infection and death rates in rural areas have surpassed those of nonrural areas.^45^, ^46^, ^47^ Further, vaccination uptake has been slower in rural areas than urban ones; by mid‐April 2021, 42% of rural [State] had been vaccinated against COVID‐19 compared to 49.6% of urban [State].^42^ A larger percentage of rural [State] residents traveled outside their county of residence to receive the vaccine compared to their urban peers. Rural residents were more likely to report they would definitely not get a COVID‐19 vaccine, which was associated with lower educational attainment and lower income.^48^ Perceptions of effectiveness, vaccine hesitancy, distance and time to receive a vaccine, and availability of vaccines all play a role.

### Strengths and limitations

This study had several strengths. LCA allowed us to identify groupings of agricultural producers and stakeholders according to patterns of perception of effectiveness and practice of COVID‐19 protective behaviors and estimate their relative population sizes. While previous studies that used LCA on COVID‐19 protective practices derived only 1 set of clusters, our study derived separate clusters for both perceptions of effectiveness and practices. Though these clusters are highly correlated, we found that there are some factors that they might not share. For example, work factors were associated with practices but not with perceptions. The large sample size enabled the detection of more distinct groups, including those in relatively smaller proportions, for instance, the social skeptics and low adherents who comprised less than 8% and 15% of the sample, respectively. It also provided more power to examine the association of these groups with demographic and health characteristics. Overall, the LCA method enabled and streamlined the discovery of associations between clusters of COVID‐19 protective practices and perceptions of effectiveness with demographic and health characteristics, which would otherwise be lost when analyzing the items individually.

Results from this study should be interpreted considering some limitations. Results were drawn from a convenience sample of agricultural producers and stakeholders recruited through agricultural organizations and commodity interest groups. A response rate could not be calculated due to this passive recruitment strategy. Given these 2 conditions, we cannot assume the results are generalizable to the broader agricultural population; however, the demographics of the sample are similar to that of the agricultural producer population in the United States.^17^ Participants were not provided a definition for rural, and designation from participants was based on their perception of residential environment and not a definition by USDA or the US Census Bureau. Additionally, distribution of the clusters derived in this study may not be same for the entire population of agricultural producers and stakeholders, and it is possible that other clusters would be obtained with a representative sample. As a cross‐sectional study, we are only able to report correlations and associations between variables rather than causal relationships. Another limitation is that behaviors were self‐reported and may have reflected social desirability bias.^49^ There are many potential influences on the use of COVID‐19 protective practices and perceptions of effectiveness, which we did not include in our survey, such as personal experience with COVID‐19 (e.g. self, family, and friends), clarity around best practices for prevention, and social pressures that regulate behavior in public.

Data collection occurred from April to June of 2020. It is unknown whether agricultural producers’ and stakeholders’ perceptions of effectiveness of COVID‐19 protective behaviors and participation in protective behaviors have changed over time. Liao et al repeated cross‐sectional surveys and observed consistent latent classes over time, but an increase in public vigilance throughout an epidemic.^50^ An additional line of inquiry is if and how individuals move across latent classes. Interventions to encourage individuals to move from low adherence groups to moderate or high adherence groups could be tested and have significant public health benefits.

### Conclusion

Overall, our results demonstrate varying levels of compliance with recommended COVID‐19 protective behaviors, and that such behaviors are associated with their perceived effectiveness and several demographic characteristics. The findings of the current study indicate that should public health campaigns focus on specific agricultural populations, rural residents, men, people under age 65, those without comorbidities, and those who report being self‐employed would be appropriate. In addition, campaigns that draw on rural community values and norms around social connections and connectedness^51^ to construct messages about COVID‐19 precautions may be effective.

## CONFLICT OF INTEREST

The authors have no conflicts of interest.

## DISCLOSURES

The authors have nothing to disclose.

## APPENDIX A

**TABLE A1:** Fit indices for latent class models

	No. of clusters	Log likelihood	df	G^2^	AIC	CAIC	BIC	SABIC	Entropy
Perceptions of effectiveness of COVID‐19 protective behaviors	2	–7,280.7	2,157	1,863.3	1,921.3	2,103.0	2,074.0	1,981.9	0.82
	3	–7,057.3	2,142	1,416.5	1,504.5	1,780.2	1,736.2	1,596.4	0.80
	4	–6,869.2	2,127	1,040.3	1,158.3	1,528.0	1,469.0	1,281.6	0.80
	5	–6,789.0	2,112	879.8	1,027.8	**1** **,491.5**	**1,417.5**	1,182.5	0.78
	6	–6,747.1	2,097	796.0	974.0	1,531.8	1,442.8	1,160.1	0.77
	7	–6,718.2	2,082	738.2	946.2	1,598.0	1,494.0	1,163.6	0.76
	8	–6,698.4	2,067	698.8	936.8	1,682.5	1,563.5	1,185.5	0.78
Participation in COVID‐19 protective behaviors	2	–10,703.5	78,067	4,261.9	4,375.9	4,733.5	4,676.5	4,495.4	0.78
	3	–10,501.5	78,038	3,858.0	4,030.0	**4,569.5**	**4,483.5**	4,210.3	0.74
	4	–10,397.7	78,009	3,650.5	3,880.5	4,601.9	4,486.9	4,121.6	0.73
	5	–10,318.2	77,980	3,491.4	3,779.4	4,682.7	4,538.7	4,081.3	0.73
	6	–10,257.5	77,951	3,370.1	3,716.1	4,801.3	4,628.3	4,078.7	0.73
	7	–10,218.3	77,922	3,291.5	3,695.5	4,962.7	4,760.7	4,119.0	0.75
	8	–10,186.8	77,893	3,228.6	3,690.6	5,139.7	4,908.7	4,174.9	0.76

Bold represent the lowest values of CAIC (Consistent Akaike Information Criterion) and BIC (Bayesian Information Criterion). These measures are used to determine which specific LCA model (e.g. two‐cluster model, three‐cluster model, four‐cluster model, etc.) best fits the data. The lower the values of CAIC and BIC, the better the model. For perceptions of effectiveness of COVID‐19 protective behaviors, the five‐cluster model had the lowest CAIC and BIC, while for practice of COVID‐19 protective behaviors, the three‐cluster model had the lowest values for these criteria.

**TABLE A2:** Tests of measurement invariance for clusters of perception of effectiveness and participation in COVID‐19 protective behaviors: comparison of fit statistics of LCA with free‐parameter measurement model and measurement invariant model, by grouping variable

LCA of perception of effectiveness of COVID‐19 protective behaviors				
Grouping variable	Free‐parameter measurement model		Measurement invariant model	
	AIC	BIC	AIC	BIC
Gender	1,348.0	2,125.9	1,281.5	1,691.5
Age	1,393.5	2,173.0	1,382.2	1,793.0
Occupation	1,524.7	2,694.0	1,382.4	1,814.3
Self‐employment	1,318.7	2,090.7	1,263.9	1,670.8
Rural/urban residence	1,310.4	2,089.8	1,264.3	1,674.2
Presence/absence of underlying health conditions	1,390.0	2,169.5	1321.8	1734.6

LCA of participation in COVID‐19 protective behaviors				
Grouping variable	Free‐parameter measurement model		Measurement invariant model	
	AIC	BIC	AIC	BIC
Gender	4,597.3	5,502.5	4,609.8	5,072.1
Age	4,894.4	5,801.4	5,042.4	5,506.5
Occupation	5,007.4	5,367.9	4,912.5	5,387.0
Self‐employment	4,769.2	5,667.6	4,740.7	5,200.3
Rural/urban residence	4,629.2	5,536.0	4,552.2	5,016.3
Presence/absence of underlying health conditions	4,786.8	5,639.8	4,779.7	5,243.7

**TABLE A3:** Odds ratios and 95% CIs for demographic and health profile using specific pairwise comparisons of clusters of perception of effectiveness of COVID‐19 protective behaviors as outcome

Factor associated	Compared to universal believer as reference outcome group			
	Personal believer	Social skeptic	Moderate believer	Social believer
Gender (male vs female)	1.55 (1.02‐2.36)	6.76 (2.62‐17.45)	4.80 (2.55‐9.04)	3.93 (1.67‐9.28)
Age (older by 10 years)	0.95 (0.94‐0.97)	0.94 (0.92‐0.95)	0.98 (0.97‐0.99)	1.02 (0.99‐1.04)
Occupation (producer vs stakeholder)	0.91 (0.52‐1.62)	3.94 (1.16‐13.41)	0.79 (0.44‐1.42)	0.90 (0.40‐2.02)
Occupation (both vs stakeholder)	2.25 (0.69‐7.33)	6.94 (1.21‐39.89)	1.28 (0.32‐5.09)	3.19 (0.73‐14.01)
Employed (yes vs no)	1.06 (0.69‐1.61)	0.92 (0.50‐1.67)	1.04 (0.66‐1.65)	1.34 (0.72‐2.49)
Residence (rural vs urban)	0.67 (0.38‐1.18)	0.55 (0.23‐1.29)	0.72 (0.39‐1.34)	0.71 (0.34‐1.46)
Health conditions (with vs without)	0.65 (0.41‐1.05)	0.70 (0.34‐1.44)	0.91 (0.58‐1.42)	0.63 (0.35‐1.11)

	Compared to personal believer as reference outcome group			
	Universal believer	Social skeptic	Moderate believer	Social believer
Gender (male vs female)	0.65 (0.42‐0.98)	4.36 (1.66‐11.44)	3.09 (1.56‐6.13)	2.54 (1.02‐6.33)
Age (older by 10 years)	1.05 (1.03‐1.07)	0.98 (0.96‐1.00)	1.03 (1.01‐1.05)	1.07 (1.04‐1.09)
Occupation (producer vs stakeholder)	1.09 (0.62‐1.94)	4.31 (1.25‐14.89)	0.87 (0.45‐1.66)	0.98 (0.42‐2.30)
Occupation (both vs stakeholder)	0.44 (0.14‐1.44)	3.08 (0.62‐15.28)	0.57 (0.16‐2.05)	1.42 (0.34‐5.87)
Employed (yes vs no)	0.95 (0.62‐1.44)	0.87 (0.47‐1.60)	0.99 (0.59‐1.64)	1.27 (0.67‐2.40)
Residence (rural vs urban)	1.49 (0.85‐2.63)	0.82 (0.33‐2.02)	1.08 (0.54‐2.17)	1.05 (0.48‐2.33)
Health conditions (with vs without)	1.53 (0.95‐2.46)	1.07 (0.49‐2.35)	1.39 (0.78‐2.46)	0.96 (0.49‐1.90)

	Compared to social skeptic as reference outcome group			
	Universal believer	Personal believer	Moderate believer	Social believer
Gender (male vs female)	0.15 (0.06‐0.38)	0.23 (0.09‐0.60)	0.71 (0.23‐2.20)	0.58 (0.17‐2.00)
Age (older by 10 years)	1.07 (1.05‐1.09)	1.02 (1.00‐1.04)	1.05 (1.03‐1.07)	1.09 (1.06‐1.12)
Occupation (producer vs stakeholder)	0.25 (0.07‐0.86)	0.23 (0.07‐0.80)	0.02 (0.06‐0.72)	0.23 (0.06‐0.89)
Occupation (both vs stakeholder)	0.14 (0.03‐0.83)	0.32 (0.07‐1.61)	0.18 (0.03‐1.16)	0.46 (0.07‐2.96)
Employed (yes vs no)	1.09 (0.60‐1.99)	1.15 (0.63‐2.12)	1.14 (0.57‐2.27)	1.46 (0.70‐3.07)
Residence (rural vs urban)	1.82 (0.77‐4.29)	1.22 (0.49‐3.02)	1.32 (0.51‐3.41)	1.29 (0.49‐3.39)
Health conditions (with vs without)	1.43 (0.69‐2.95)	0.93 (0.42‐2.05)	1.30 (0.57‐2.94)	0.90 (0.39‐2.07)

	Compared to moderate believer as reference outcome group			
	Universal believer	Personal believer	Social skeptic	Social believer
Gender (male vs female)	0.21 (0.11‐0.39)	0.32 (0.16‐0.64)	1.41 (0.46‐4.36)	0.82 (0.28‐2.38)
Age (older by 10 years)	1.02 (1.01‐1.04)	0.97 (0.95‐0.99)	0.95 (0.94‐0.97)	1.04 (1.01‐1.06)
Occupation (producer vs stakeholder)	1.26 (0.71‐2.27)	1.16 (0.60‐2.21)	4.98 (1.39‐17.81)	1.13 (0.47‐2.73)
Occupation (both vs stakeholder)	0.78 (0.20‐3.11)	1.76 (0.49‐6.37)	5.43 (0.86‐34.19)	2.50 (0.53‐11.69)
Employed (yes vs no)	0.96 (0.61‐1.52)	1.01 (0.61‐1.69)	0.88 (0.44‐1.76)	1.29 (0.65‐2.53)
Residence (rural vs urban)	1.38 (0.75‐2.55)	0.93 (0.46‐1.86)	0.76 (0.29‐1.96)	0.97 (0.41‐2.30)
Health conditions (with vs without)	1.10 (0.71‐1.72)	0.72 (0.41‐1.28)	0.77 (0.34‐1.74)	0.69 (0.36‐1.33)

	Compared to social believer as reference outcome group			
	Universal believer	Personal believer	Social skeptic	Moderate believer
Gender (male vs female)	0.25 (0.11‐0.60)	0.39 (0.16‐0.98)	1.72 (0.50‐5.92)	1.22 (0.42‐3.54)
Age (older by 10 years)	0.98 (0.96‐1.01)	0.94 (0.91‐0.96)	0.92 (0.00‐0.95)	0.96 (0.94‐0.99)
Occupation (producer vs stakeholder)	1.12 (0.50‐2.52)	1.02 (0.44‐2.40)	4.40 (1.13‐17.15)	0.88 (0.37‐2.13)
Occupation (both vs stakeholder)	0.31 (0.07‐1.38)	0.71 (0.17‐2.93)	2.18 (0.34‐14.00)	0.40 (0.09‐1.88)
Employed (yes vs no)	0.75 (0.40‐1.39)	0.79 (0.42‐1.49)	0.68 (0.33‐1.44)	0.78 (0.40‐1.53)
Residence (rural vs urban)	1.42 (0.68‐2.94)	0.95 (0.43‐2.10)	0.78 (0.30‐2.05)	1.03 (0.43‐2.43)
Health conditions (with vs without)	1.60 (0.90‐2.83)	1.04 (0.53‐2.06)	1.11 (0.48‐2.58)	1.45 (0.75‐2.78)

**TABLE A4:** Odds ratios and 95% CIs for demographic and health profile using specific pairwise comparisons of clusters of practice in COVID‐19 protective behaviors as outcome

	Compared to high adherent as reference outcome group	
Factor associated	Moderate adherent	Low adherent
Gender (male vs female)	3.21 (2.05‐5.02)	12.69 (3.44‐46.84)
Age (older by 10 years)	0.96 (0.95‐0.98)	0.96 (0.95‐0.98)
Occupation (producer vs stakeholder)	0.85 (0.53‐1.38)	1.15 (0.51‐2.57)
Occupation (both vs stakeholder)	0.96 (0.38‐2.43)	1.05 (0.25‐4.34)
Employed (yes vs no)	1.23 (0.85‐1.79)	1.95 (1.08‐3.52)
Residence (rural vs urban)	1.35 (0.81‐2.26)	2.33 (0.89‐6.10)
Health conditions (with vs without)	0.72 (0.49‐1.04)	0.50 (0.26‐0.93)

	Compared to moderate adherent as reference outcome group	
	High adherent	Low adherent
Gender (male vs female)	0.31 (0.20‐0.49)	3.95 (0.99‐15.75)
Age (older by 10 years)	1.04 (1.02‐1.05)	1.00 (0.98‐1.01)
Occupation (producer vs stakeholder)	1.18 (0.73‐1.90)	1.35 (0.58‐3.12)
Occupation (both vs stakeholder)	1.05 (0.41‐2.66)	1.10 (0.26‐4.57)
Employed (yes vs no)	0.81 (0.56‐1.17)	1.58 (0.85‐2.93)
Residence (rural vs urban)	0.74 (0.44‐1.23)	1.72 (0.60‐4.93)
Health conditions (with vs without)	1.40 (0.96‐2.04)	0.69 (0.35‐1.38)

	Compared to low adherent as reference outcome group	
	High adherent	Moderate adherent
Gender (male vs female)	0.08 (0.02‐0.29)	0.25 (0.06‐1.01)
Age (older by 10 years)	1.04 (1.02‐1.06)	1.00 (0.99‐1.02)
Occupation (producer vs stakeholder)	0.87 (0.39‐1.95)	0.74 (0.32‐1.71)
Occupation (both vs stakeholder)	0.95 (0.23‐3.93)	0.91 (0.22‐3.78)
Employed (yes vs no)	0.51 (0.28‐0.93)	0.63 (0.34‐1.18)
Residence (rural vs urban)	0.43 (0.16‐1.13)	0.58 (0.20‐1.66)
Health conditions (with vs without)	2.02 (1.08‐3.79)	1.44 (0.72‐2.88)
